# The use of artificial intelligence in the prevention and management of bleeding disorders: a systematic review

**DOI:** 10.3389/fmed.2025.1606788

**Published:** 2025-10-09

**Authors:** Fathima Raahima Riyas Mohamed, Ziyad Aldabbagh, Wael Kalou, Khaled Hamsho, Anwar Aldabbagh, Adel Kalou, Muhammad Raihan Sajid

**Affiliations:** College of Medicine, Alfaisal University, Riyadh, Saudi Arabia

**Keywords:** artificial intelligence, machine learning, bleeding disorders, hemophilia A, von Willebrand disease, immune thrombocytopenia, predictive models

## Abstract

**Background:**

Bleeding disorders, including hemophilia, von Willebrand disease (VWD), and immune thrombocytopenia (ITP), pose significant diagnostic and therapeutic challenges due to their heterogeneous presentations and complex underlying mechanisms. Traditional diagnostic methods rely on clinical assessments and laboratory tests, which can be time-consuming and prone to misdiagnosis, particularly in resource-limited settings. Artificial intelligence (AI) has emerged as a transformative tool in healthcare, leveraging machine learning (ML) algorithms and predictive analytics to enhance diagnostic accuracy, risk stratification, and personalized treatment approaches.

**Objective:**

This systematic review explores the role of AI in the prevention, diagnosis, and management of bleeding disorders. Specifically, it assesses AI-driven models in identifying key predictors, optimizing risk assessment, and improving treatment outcomes.

**Methods:**

A comprehensive literature search was conducted across major databases following PRISMA guidelines. Studies were selected based on their focus on AI applications in bleeding disorders, particularly those utilizing ML models such as Random Forest, XGBoost, LightGBM, and deep learning techniques. The risk of bias was evaluated using the ROBINS-E and RoB 2 tools.

**Results:**

Twelve studies met the inclusion criteria, demonstrating the efficacy of AI models in bleeding disorder management. Genetic markers, such as Factor VIII gene mutations and von Willebrand factor variants, enable early disease classification and severity prediction. Laboratory biomarkers, including baseline factor VIII activity, platelet count, and coagulation profiles, enhance risk assessment for bleeding complications. Clinical history variables, such as prior bleeding events, anticoagulant use, infection status, and comorbidities, support personalized treatment strategies. Additionally, demographic and environmental factors, including age, sex, healthcare utilization patterns, and socioeconomic status, refine predictive models for undiagnosed cases.

**Conclusion:**

The integration of these variables into AI-driven models has demonstrated superior diagnostic accuracy compared to traditional methods, facilitating early detection, individualized treatment planning, and improved patient outcomes. However, challenges such as dataset fragmentation, model interpretability, and limited external validation hinder widespread clinical adoption. AI-driven approaches have the potential to revolutionize bleeding disorder management by advancing precision medicine, optimizing healthcare resources, and promoting equitable access to high-quality care.

## Introduction

Bleeding disorders are a heterogeneous group of hereditary and acquired conditions characterized by impaired hemostasis, resulting in excessive or prolonged bleeding (1). Hemostasis is a tightly regulated process that prevents blood loss after vascular injury, involving three primary phases: vascular constriction, platelet aggregation to form a temporary plug, and activation of the coagulation cascade, which culminates in fibrin mesh formation to stabilize the clot (1–3). Disruptions in any of these processes–caused by platelet dysfunction (4, 5), clotting factor deficiencies (6, 7), fibrinolytic abnormalities (8, 9), or vascular defects (10)–can lead to uncontrolled bleeding, spontaneous hemorrhage, and life-threatening complications.

Bleeding disorders are broadly classified into platelet disorders, coagulation factor deficiencies, and vascular abnormalities (11). Platelet disorders, such as Glanzmann Thrombasthenia, Bernard-Soulier syndrome, and immune thrombocytopenic purpura (ITP), impair platelet adhesion, aggregation, or survival (12). Coagulation factor deficiencies include hemophilia A (factor VIII deficiency) (6), hemophilia B (factor IX deficiency) (13), and von Willebrand disease (VWD), which affects von Willebrand factor (VWF) and impairs clot formation (14). Rare factor deficiencies–including fibrinogen, prothrombin, and factors V, VII, XI, and XIII–also contribute to abnormal bleeding (15). Vascular abnormalities, such as Ehlers-Danlos syndrome and hereditary hemorrhagic telangiectasia, increase vessel fragility and bleeding risk (16, 17).

Clinically, bleeding disorders present along a spectrum depending on severity and affected pathway (18). Mild cases may show easy bruising, frequent epistaxis, or prolonged bleeding after minor injuries, whereas severe cases can cause spontaneous joint (hemarthrosis) or muscle bleeds, gastrointestinal hemorrhage, and intracranial bleeding (1, 18, 19). Recurrent bleeding, particularly hemarthrosis, can lead to joint damage, disability, and reduced quality of life (20).

Despite advances in understanding, diagnosing and managing bleeding disorders remain challenging, especially in resource-limited settings (21, 22). Diagnosis relies on clinical history, bleeding assessment tools (BATs), and specialized laboratory tests, including platelet function assays, clotting factor measurements, and genetic testing (22). Access to these tools varies widely; globally, only 6.3% of individuals with inherited bleeding disorders receive a diagnosis, with rates below 10% in low- and middle-income countries (LMICs) compared to 55% in high-income countries (HICs) (23). Limited laboratory infrastructure, high assay costs, and variable symptom presentation contribute to underdiagnosis and misdiagnosis (24, 25).

Artificial intelligence (AI) offers promising solutions to these challenges. AI encompasses machine learning (ML) and deep learning techniques that analyze complex datasets to identify patterns, make predictions, and support decision-making (26). In clinical medicine, AI has improved diagnostic accuracy, predicted outcomes, and personalized treatment (26, 27). ML algorithms, such as Random Forest and Extreme Gradient Boosting (XGBoost), excel at detecting non-linear relationships in high-dimensional data, making them suitable for predictive modeling (28). In fields such as radiology, pathology, and endoscopy, AI has enhanced diagnostic efficiency, reduced errors, and optimized care delivery (29–31).

In bleeding disorders, AI can similarly improve diagnosis by integrating clinical and laboratory data, predict bleeding risks through advanced modeling, and individualize treatment to optimize outcomes (32). It may also identify novel therapeutic targets via genomic and proteomic analyses (33). However, AI adoption in bleeding disorders lags behind other domains, likely due to disease rarity, fragmented datasets, and limited systematic evaluation (34).

This systematic review aims to comprehensively synthesize the existing evidence on the application of artificial intelligence (AI) in the diagnosis, treatment, and prevention of bleeding disorders, an area that has received less attention compared to oncology or cardiology. The primary objectives are to evaluate the potential of AI to enhance diagnostic accuracy and facilitate early intervention, assess AI-driven methodologies for predicting bleeding risks and optimizing treatment protocols, and explore AI’s role in identifying novel therapeutic targets for bleeding disorders. Additionally, this review seeks to identify the current limitations and challenges in integrating AI into the management of bleeding disorders, while also proposing future directions for its application in this field. By systematically appraising the available evidence using established risk-of-bias tools, it clarifies both the potential and the limitations of current models. In particular, it draws attention to persistent gaps such as the lack of prospective validation, minimal use of external datasets, and the underrepresentation of low-resource settings–issues that are essential to address for future clinical adoption.

## Methodology

This systematic review followed PRISMA-2020 guidelines (Preferred Reporting Items for Systematic Reviews and Meta-Analyses) to maintain a thorough and consistent approach across all stages, from literature search to data synthesis (35). The study framework was guided by the PICOS model (Population, Intervention, Comparison, Outcomes, and Study Designs), with inclusion and exclusion criteria explicitly defined to align with the research objectives, as outlined in Table 1.

**TABLE 1:** PICOS framework.

PICOS	Description
P (participants)	Patients with bleeding disorders (such as hemophilia, von Willebrand disease, and other coagulopathies).
I (intervention)	Use of artificial intelligence (AI) techniques (for instance, machine learning, deep learning, and predictive algorithms) in the diagnosis, prevention, and management of bleeding disorders.
C (comparisons)	Traditional approaches or no AI intervention in the prevention and management of bleeding disorders, or usual care.
O (outcomes)	Improved patient outcomes, including reduced bleeding episodes, better management of bleeding events, optimized dosing of treatments, early prediction and prevention of bleeding risks, and overall enhancement in quality of life and healthcare efficiency.
S (study designs)	Human studies.

### Literature search

A detailed search strategy was implemented to locate relevant studies exploring the use of Artificial Intelligence (AI) in preventing and managing bleeding disorders. The search covered prominent databases, including PubMed, Science Direct, Google Scholar, and Wiley. Keywords and Medical Subject Headings (MeSH) terms were combined using Boolean operators (AND/OR) to enhance the search precision. The query included terms such as: (Bleeding Disorders OR Hemophilia OR Von Willebrand Disease OR Coagulopathy OR Clotting Disorders OR Thrombocytopenia OR Hemostasis disorders) AND (Artificial Intelligence OR AI OR ML OR Machine Learning OR Deep Learning OR Predictive Algorithms OR Predictive Modeling OR Computer-Assisted Diagnosis). Searches were finalized on 02/12/2024, with citations managed through Rayyan software to eliminate duplicates and facilitate initial screening (36).

### Inclusion and exclusion criteria

The inclusion criteria for this systematic review were established using the PICO framework to ensure methodological rigor. Eligible studies focused on populations diagnosed with bleeding disorders, including hemophilia, von Willebrand disease, and other coagulopathies. The intervention of interest was the application of Artificial Intelligence (AI) techniques, such as machine learning, deep learning, and predictive algorithms, in the diagnosis, prevention, and management of bleeding disorders. Studies were required to include a comparison with traditional approaches, usual care, or no AI-based intervention. The review prioritized studies reporting outcomes related to improved patient care, including reductions in bleeding episodes, enhanced management of bleeding events, optimized dosing of treatments, early prediction and prevention of bleeding risks, and overall improvements in quality of life and healthcare efficiency. Only human studies employing randomized controlled trials, observational designs, cross-sectional studies, or cohort studies were considered for inclusion.

Exclusion criteria were defined to maintain the focus on high-quality, peer-reviewed evidence. Non-peer-reviewed literature, such as editorials, opinion pieces, conference reports, or abstracts, was excluded, along with case reviews, case series, review articles, and case reports. Studies written in languages other than English and those involving animal models were also excluded. These criteria were applied to ensure that the review synthesized robust and relevant evidence regarding the role of AI in the prevention and management of bleeding disorders.

### Literature screening

The initial screening process was conducted systematically, beginning with a review of article titles, followed by an evaluation of abstracts. Each title and abstract were carefully assessed against the predefined inclusion and exclusion criteria. In the subsequent stage, full-text articles were subjected to a detailed review to ensure they addressed the use of Artificial Intelligence (AI) in the diagnosis, prevention, or management of bleeding disorders. Particular attention was given to studies that provided adequate scientific detail on AI techniques, their applications, and their impact on patient outcomes. This rigorous three-step screening process ensured the inclusion of studies that would contribute to a comprehensive and relevant dataset for understanding the role of AI in improving the diagnosis, prevention, and management of bleeding disorders.

### Data extraction

Data were systematically extracted from each included study using a structured Microsoft Excel form to ensure a comprehensive and accurate capture of key information. The extracted data included details on study design, country of origin, and total sample size, as well as participant characteristics such as gender, age, and ethnicity. Inclusion and exclusion criteria were documented, encompassing symptoms, medical history, diagnostic methods, and other relevant factors. Specific information related to model development was also recorded, including data sources used, training and testing processes, and model performance metrics such as accuracy, precision, and sensitivity.

Additional variables extracted included key predictors identified by the models, the number of undiagnosed cases, and the main characteristics of these undiagnosed cases. Study limitations, outcome definitions, data processing methods, exploratory data analysis findings, and validation strategies were meticulously noted. Details on treatments administered to patients were also collected. This structured and systematic approach to data extraction ensured the inclusion of all relevant variables necessary for a comprehensive evaluation of the role of Artificial Intelligence (AI) in the diagnosis, prevention, and management of bleeding disorders.

### Risk of bias assessment

To assess the risk of bias in the included studies, we utilized a variety of validated tools, each specifically designed for different study types, to ensure a thorough and consistent evaluation.

For observational studies, the Risk of Bias in Non-randomized Studies - of Exposures (ROBINS-E) tool was employed. This tool evaluates bias across multiple domains, including confounding, participant selection, exposure classification, deviations from intended exposures, missing data, outcome measurement, and reporting selection. Each observational study was reviewed using these criteria, enabling a detailed assessment of potential biases specific to non-randomized studies. The domains were rated as low risk (L), moderate risk (M), serious risk (S), critical risk (C), or no information (NI) (37).

The risk of bias assessment for randomized controlled trials (RCTs) included in this review was conducted using the Cochrane Risk of Bias 2 (RoB 2) tool, a rigorous and widely accepted framework for evaluating the methodological quality of RCTs. The assessment focused on five key domains: bias arising from the randomization process, bias due to deviations from intended interventions, bias due to missing outcome data, bias in the measurement of outcomes, and bias in the selection of the reported result. Each domain was systematically evaluated, and studies were rated as having a low risk of bias, some concerns, or a high risk of bias based on predefined criteria (38).

### Data synthesis

A quantitative meta-analysis was not performed due to substantial heterogeneity across included studies. Variability existed in AI model architectures (such as Random Forest, XGBoost, Graph Neural Networks), predictor variables (genetic, clinical, laboratory, environmental), outcome definitions (for instance, short-term bleeding risk, inhibitor development, disease severity classification), and performance metrics (accuracy, AUROC, F1-score, PPV). This heterogeneity precluded valid statistical pooling, so a narrative synthesis approach was adopted.

## Results

### Study selection

The initial phase of screening the identified studies involved reviewing their Titles and Abstracts to assess relevance based on the defined PICOS criteria for this systematic review. The search yielded 2,927 records, which were imported into Rayyan software to streamline and organize the screening process. Rayyan automatically identified and removed 161 duplicate entries, leaving 2,766 unique records for evaluation.

The first stage of screening focused on assessing Titles and Abstracts based on the pre-established inclusion and exclusion criteria. This resulted in the exclusion of 2,714 studies that did not align with the review’s core focus on the use of Artificial Intelligence (AI) in the prevention and management of bleeding disorders. In the next phase, a more in-depth review was conducted for the remaining 52 abstracts. This stage involved examining the relevance of each study to AI applications in risk prediction, diagnostic advancements, and treatment optimization for bleeding disorders. Studies that did not directly address these topics were excluded, leaving 21 articles for full-text evaluation.

The final phase involved a careful full-text assessment to ensure compliance with the inclusion criteria. Nine studies were excluded due to inadequate focus or lack of relevant data, leaving a total of 12 studies for inclusion in the systematic review. This rigorous selection process ensured a reliable foundation for understanding AI applications in bleeding disorder management.

**FIGURE 1:**
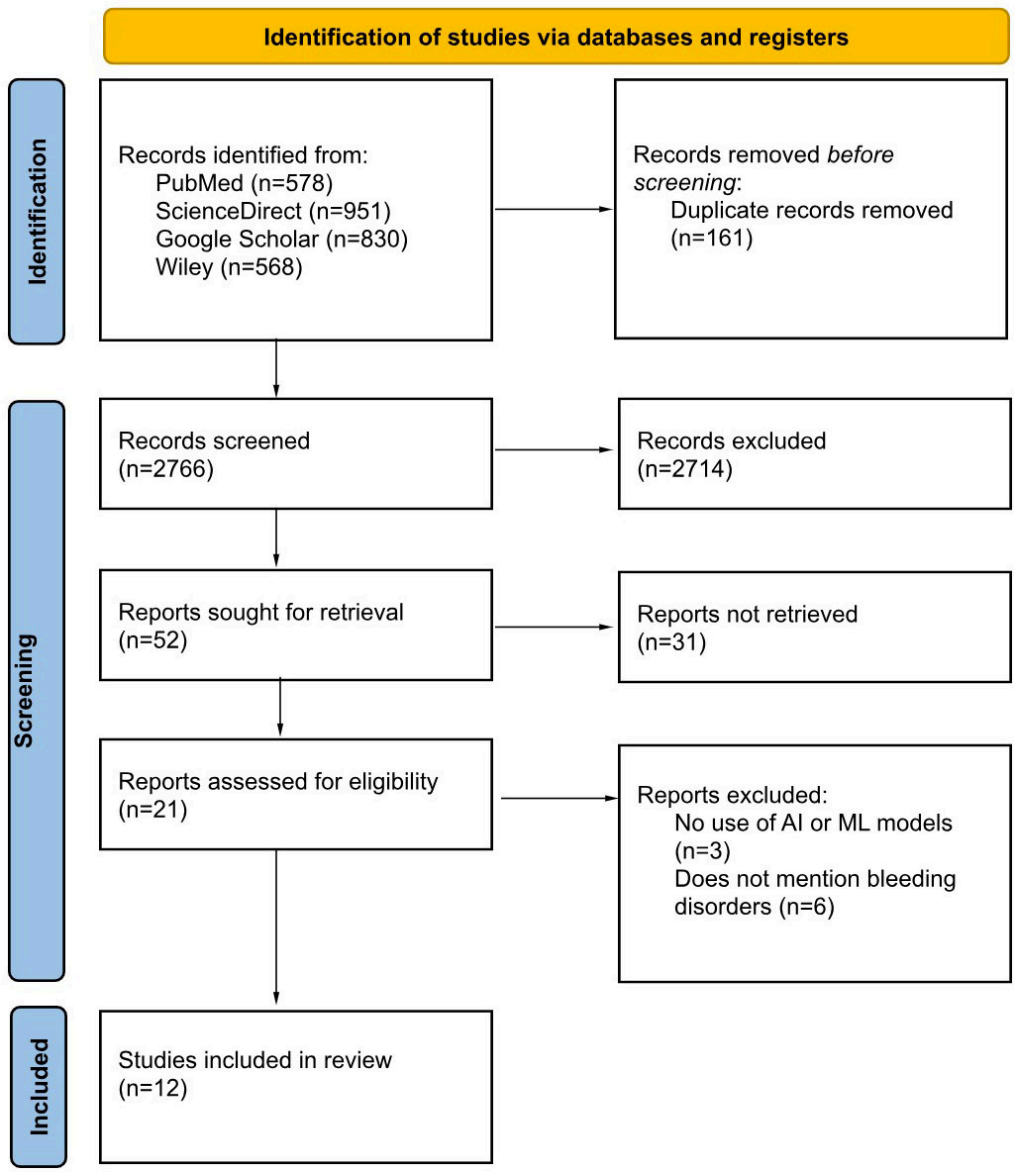
Preferred reporting items for systematic reviews and meta-analyses (PRISMA) diagram demonstrating search strategy.

To provide a clear overview of the multi-stage review process and enhance methodological transparency, a PRISMA flowchart (Figure 1) was created, illustrating the progression from the initial search to the final selection of studies included in the review (25).

### Risk of bias assessment

The risk of bias assessment for the included studies was conducted in two stages: separately for non-randomized studies and randomized-control studies.

The risk of bias assessment for the studies, presented in Table 2, was conducted using the ROBINS-E tool, which evaluates the quality of non-randomized studies based on seven domains: risk of bias due to confounding, bias arising from the measurement of exposure, bias in the selection of participants, bias due to post-exposure interventions, bias due to missing data, bias arising from the measurement of outcomes, and bias in the selection of reported results (37).

**TABLE 2:** Risk of bias analysis using the ROBINS-E tool - for non-randomized studies.

Study	Item and score							Overall risk
	Risk of bias due to confounding	Risk of bias arising from measurement of exposure	Risk of bias in selection of participants	Risk of bias due to post-exposure intervention	Risk of bias due to missing data	Risk of bias arising from measurement of outcome	Risk of bias in selection of reported result	
An et al. (39)	Moderate risk	Low risk	N/A	N/A	Low risk	Low risk	Low risk	Moderate risk
Ferreira et al. (49)	Low risk	Low risk	Low risk	N/A	Moderate risk	Moderate risk	Low risk	Moderate risk
Hu et al. (40)	Moderate risk	Moderate risk	Low risk	N/A	High risk	Moderate risk	Moderate risk	High risk
Lopes et al. (41)	Moderate risk	Moderate risk	Low risk	N/A	Moderate risk	Moderate risk	Low risk	High risk
Lyons et al. (42)	Moderate risk	Moderate risk	Low risk	N/A	Moderate risk	Low risk	Low risk	Moderate risk
Miah et al. (43)	Moderate risk	Low risk	Low risk	N/A	Moderate risk	Low risk	Low risk	Moderate risk
Singh et al. (44)	Moderate risk	Low risk	Low risk	N/A	Moderate risk	Low risk	Moderate risk	Moderate risk
Rawal et al. (45)	Moderate risk	Moderate risk	N/A	N/A	Moderate risk	Moderate risk	Moderate risk	High risk
Sidonio Jr et al. (50)	Low risk	Moderate risk	Low risk	N/A	Low risk	Low risk	Low risk	Low risk
Aleksic et al. (46)	Moderate risk	Low risk	Moderate risk	N/A	Moderate risk	Low risk	Moderate risk	Moderate risk
Sidonio Jr et al. (47)	Moderate risk	Low risk	Low risk	Low risk	Moderate risk	Low risk	Moderate risk	Moderate risk

N/A, not available.

In the domain of risk of bias due to confounding, the majority of the included studies were rated as having moderate risk, likely due to incomplete adjustment for confounding factors or unclear reporting of control strategies (39–48). However, studies such as Ferreira et al., 202, and Sidonio Jr et al., achieved a low-risk rating, suggesting more rigorous confounder control (49, 50).

For bias arising from the measurement of exposure, most studies demonstrated a low risk, indicating reliable assessment methods (39, 43, 44, 46–48). However, moderate concerns were noted in some studies possibly due to measurement inaccuracies or unclear exposure definitions (40–42, 45, 50). The domain of risk of bias in the selection of participants was generally low risk, except for Aleksić et al., which was rated as moderate risk, potentially due to a small sample size from a single center that might introduce bias (46).

In the domain of bias due to missing outcome data, moderate risk was observed in several studies (41–49), often due to incomplete follow-up or inadequate reporting strategies. Notably, Hu et al., was rated as high risk (40), whereas studies like An et al., and Sidonio Jr et al., (39, 50) demonstrated low risk, indicating strong data management practices.

For bias arising from the measurement of outcomes, most studies were categorized as low risk, while studies such as Hu et al., Lopes et al., Ferreira et al., and Rawal et al., received a moderate risk rating, suggesting potential inconsistencies in outcome measurement (40, 41, 45, 49). Lastly, in the bias of selection of reported results, Rawal et al., Hu et al., Singh et al., Sidonio Jr et al., and Aleksić et al., were rated as moderate risk, indicating possible selective reporting that could exaggerate findings or omit key outcomes (40, 44–47). This analysis underscores the varying degrees of bias present across studies, emphasizing the need for careful interpretation, particularly for those with high overall risk ratings.

The risk of bias assessment for the randomized controlled trial (RCT) included in this review was conducted using the Cochrane Risk of Bias 2 (RoB 2) tool, which evaluates the quality of RCTs across five key domains: bias arising from the randomization process, bias due to deviations from intended interventions, bias due to missing outcome data, bias in the measurement of outcomes, and bias in the selection of the reported result (38). The detailed results of this assessment are presented in Table 3.

**TABLE 3:** Risk of bias analysis using the rob 2 tool - for randomized control trials.

Study	Item and score					Overall risk
	Bias arising from the randomization process	Bias due to deviations from intended interventions	Bias due to missing outcome data	Bias in measurement of the outcome	Bias in selection of the reported result	
Chowdary et al. (48)	Moderate risk	Moderate risk	Low risk	Low risk	Low risk	Moderate risk

The study demonstrates a moderate overall risk of bias (48). While baseline characteristics were comparable across groups, the randomization process and allocation concealment were not explicitly detailed, introducing potential bias. The trial’s open-label design further contributes to performance bias, as neither participants nor personnel were blinded; however, this is mitigated by the objective nature of the primary outcome, annualized bleeding rate (ABR). Missing outcome data were handled appropriately by scaling bleed counts during the available follow-up period, reducing the risk of attrition bias. Measurement of outcomes was objective and unlikely to be influenced by assessors, given the nature of the data collected. Lastly, the study appears to have reported all predefined outcomes without evidence of selective reporting. Despite these strengths, the lack of blinding and insufficient detail on randomization contribute to a moderate risk of bias overall.

### Study characteristics

This systematic review encompassed 12 studies investigating the application of Artificial Intelligence (AI) in bleeding disorders. The studies employed diverse methodologies, including retrospective and prospective cohort studies, randomized controlled trials, and feasibility studies. Conducted in geographically diverse settings, including the United States, United Kingdom, Spain, Germany, Brazil, Japan, Serbia, India, and China, the research provided a global understanding of AI’s role in bleeding disorder management. Observation periods varied significantly, with retrospective studies analyzing years of electronic health record (EHR) data and prospective trials evaluating AI interventions over months. Refer to Table 4 for a brief summary of all included studies.

**TABLE 4:** Summary of included studies.

Study	Country	Population			Bleeding disorder studied	Exclusion criteria	Data source	Predictors identified	Model types	Performance metrics	Key results
		Sample size	Gender	Age							
An et al. (39)	China	Retrospective cohort: 2094 Prospective cohort: 1097	37.75% M and 62.25% F in the retrospective cohort	54 Y (median)	ITP	Secondary ITP. Inconsistent diagnostic criteria.	Electronic medical records	Infection, uncontrolled diabetes, Age, ITP type, cardiovascular disease, low absolute lymphocyte count, skin and mucosa bleeding, initial platelet (PLT) count, low platelet (PLT) count (<20 × 10^9^), disease duration	Random Forest (RF), XGBoost	RF achieved highest AUC. Retrospective cohort 0.89; Prospective cohort (inpatients) 0.82, (outpatients) 0.74.	Prediction and prevention of critical bleeding events. Outcomes like intracranial hemorrhage were targeted.
Miah et al. (43)	UK	150 patients (100 ITP patients from the UK Adult ITP Registry and 50 non-ITP patients from a general outpatient clinic)	ITP patients: M = 53%, F = 47% non-ITP patients: M = 58%, F = 42%	29–106 Y (ITP patients) and 25–89 Y (non-ITP patients)	ITP	Evidence of other causes of thrombocytopenia (such as liver disease, myelodysplasia). Presence of abnormal blood test results not characteristic of ITP.	UK Adult ITP Registry. Non-acute outpatient clinic data from Barts health NHS trust.	Blood platelet count	Logistic Regression, support vector machine, k-nearest neighbor, decision tree, random forest	Random forest achieved 100% accuracy.	Highlighted ITP cases based on simple blood tests. Accurate outcomes with limited diversity.
Chowdary et al. (48)	UK, Spain, USA, Germany	166	100% M	30.5 Y (±12.3 Y)	Hemophilia A	Patients who received on-demand treatment were excluded. Data was excluded for patients who did not meet the 90-day prophylaxis exposure criterion.	Data from pathfinder 2 trial (phase III)	Cumulative bleed count, baseline Von Willebrand factor level, Mean factor VIII at 30 min	Penalized logistic regression, random forest	Best-performing model (random forest) 0.785.	Adjusted prophylaxis regimens based on bleeding predictions. Outcomes measured by ABR (annual bleed rate).
Ferreira et al. (49)	Brazil, Japan	626 Hemophilia A cases with single-point, non- synonymous mutations (344 alanine mutations in the A2 and C2 domains of FVIII)	N/A	N/A	Hemophilia A	Conflicting or ambiguous phenotype classifications (such as, “Mild/Moderate”) and misidentified mutations (such as, incorrect residue positions)	FVIII mutation databases (EAHAD and CHAMP), structural data from AlphaFold2	Buried and conserved residues in the FVIII structure	Graph attention networks (GAT), SHADOW-GNN architectures	Performance: 69%–70% accuracy for severity prediction (via F1 score).	Enhanced understanding of mutation effects on hemophilia severity. Outcomes included functional validation using assays.
Hu et al. (40)	USA	23,000 individuals in the ATHN dataset; ATHN 7 subset include	N/A	N/A	Hemophilia A	Participants with significant missing data in key variables.	ATHN dataset and ATHN 7 study subset	N/A	K-nearest neighbors (KNN), XGBoost, random forest, CatBoost, logistic regression, support vector machine, and others	CatBoost achieved 67% accuracy; random forest 79% for target joint prediction.	Predictions for target joint development and inhibitor outcomes. Focused on bleeding event patterns.
Lopes et al. (41)	Brazil	Training set: 443 instances of non-synonymous mutations. Testing and validation: included over 344 patients with von Willebrand disease were excluded from specific therapy-based criteria. mutations from alanine scanning experiments and ∼1000 from CHAMP	N/A	N/A	Hemophilia A	Mutations outside coding regions. Instances with ambiguous diagnostic or activity data.	Data was curated from the EAHAD and CHAMP databases and structural protein information from the PDB (2R7E structure)	Mutation location (Buried residues, conserved regions), Network centrality	Supervised ML classifiers (such as, decision tree, random forest, SVM, naïve bayes, XGBoost)	Accuracy: 66%–87%. Predictions aligned with *in vitro* results.	Provided insights for mutation severity classification. Outcomes focused on mutation impact.
Lyons et al. (42)	USA	2,252 patients in the screening cohort; 400 patients underwent medical record abstraction.	Screening cohort: M = 81.2% Abstraction cohort: M = 77.3%	Screening cohort: 30.7 Y abstraction cohort: 34.7 Y (mean)	Hemophilia A	Patients with von Willebrand disease were excluded from specific therapy-based criteria.	Healthcore integrated research database (HIRD)	Male sex, factor VIII therapy, Hemophilia-related healthcare utilization	Lasso logistic regression with 20-fold cross-validation, Generalized boosted modeling	AUC: 0.966. Sensitivity: 94.7%.	Accurate identification of probable hemophilia A cases. Focused on healthcare utilization patterns.
Singh et al. (44)	India	7784 mutations (EAHAD dataset), 6286 analyzed	M = 100%	N/A	Hemophilia A	N/A	EAHAD database.	Mutation type, effect, position	k-nearest neighbors (KNN), adaBoost, support vector machine (SVM), random forest (RF)	RF (PSM) accuracy: 73.96%, SVM (PSM): 73.86%	Mutation severity classification based on features
Rawal et al. (45)	USA	940		Infants to older adults	Hemophilia A	Patients with incomplete variable data for ML modeling or unknown drug treatments.	Phenotypic and genomic data from the MLOF repository; additional data included human leukocyte antigen (HLA) typing and derived biological variables	Baseline factor VIII activity, foreign peptide-HLA binding affinities (mean and minimum), factor VIII mutation type	Random forest (RF), light gradient boosting machine (LGBM), logistic regression (LR), CatBoost, and others	Accuracy: ∼0.7354 (F1 score).	Accurate predictions for inhibitor status in hemophilia A. Outcomes measured inhibitor-negative probabilities.
Sidonio Jr et al. (47)	USA	Diagnosed = 10,420 Undiagnosed = 507,668	M = 14% (undiagnosed set), 28% (diagnosed set) F = 86% (undiagnosed set), 72% (diagnosed set)	The majority of best-fit patients were <46 years old.	VWD/Muco-cutaneous bleeding disorders	Diagnosed Hemophilia A Use of Anti-coagulants Patients with primary qualitative platelet disorders Patients without at least 24 months of continuous enrollment in a health plan.	IMS pharMetrics plus database (2006–2015)	Number of procedures to treat bleeding, Age, total number of bleeding events, Gastrointestinal (GI) bleeds, emergency room (ER) visits, sex	Unary predictive model using positive-unlabeled learning	Best-fit patients: 83% PPV Good-fit patients: 75% PPV	The model identified 3,318 best-fit and 37,163 good-fit undiagnosed cases.
Sidonio Jr et al. (50)	USA	Diagnosed: 5981, Undiagnosed: 4869518	M = 42% (N = 20,439) F = 58% (N = 28,463)	M = 21 Y F = 33 Y (mean)	VWD	1 + bleeding claim in 2 years prior to index date for hemophilia A/acquired hemophilia A, aortic stenosis, extracorporeal membrane oxygenation, or ventricular assist devices. 1 + hemophilia A or acquired hemophilia diagnosis claim. 1 + coagulation disorders claims or other conditions. 2 + diagnosis claims of less relevant general bleeding disorder (menstrual, genitourinary, or digestive bleeds; anemia, unspecified)	Komodo health comprehensive dataset	Heavy menstrual bleeding (HMB) in females, frequent medical procedures, hospitalizations, and emergency room visits in males	Random forest, neural network, conditional forest, gradient boosting machine	Accuracy: Males 85%, Females 84% Sensitivity: Males 77%, Females 73%	Early identification of VWD cases. Outcomes targeted appropriate treatment initiation.
Aleksic et al. (46)	Serbia	96	M = 79.2%, F = 20.8%	56.99 ± 11.46 Y	Cirrhosis with variceal bleeding risk	Baseline and time series clinical data from ICU patients.	Clinical Center of Niš	Child-pugh score, platelet count, Esophageal varices	Naive bayes, J48, LogitBoost, PART	LogitBoost accuracy: 98%	Effective screening for bleeding risk in cirrhotic patients

### Population and demographics

The studies reviewed included diverse populations with varying demographic and clinical characteristics, reflecting the heterogeneous nature of bleeding disorders. The sample sizes ranged widely, from small cohorts with 96 participants (46) to extensive cohorts with over 23,000 individuals (40, 47, 50). Age distribution varied significantly across studies, with some focusing on pediatric populations (45) and others targeting older adults. Gender representation was often influenced by the disorder under investigation. Studies focusing on hemophilia A predominantly included males, given the X-linked inheritance of the condition. For example, the Chowdary et al., study consisted entirely of males (48), while studies on von Willebrand disease (VWD) and ITP included a more balanced gender distribution, such as Miah et al., which reported 53% male and 47% female participants among ITP patients (43).

Ethnic diversity was considered in some studies, particularly those conducted in multiethnic settings like the United States. For instance, Hu et al., analyzed data from the ATHN dataset, capturing Hispanic and non-Hispanic populations (40), while studies like Rawal et al., included racial categories such as White, Black, and Asian participants (48).

### Machine learning models in bleeding disorder prediction and management

The studies employed a wide variety of machine learning algorithms, reflecting the complexity of bleeding disorders and their diverse datasets. Supervised learning models were the most commonly used, including Random Forest (39–41, 43–45, 50), XGBoost (40, 41), Gradient Boosting Machines (45, 50), CatBoost (40, 45), and Support Vector Machines (SVM) (41, 43, 44). These algorithms excelled in predictive and classification tasks, such as forecasting bleeding risks, identifying high-risk mutations, and optimizing prophylactic treatment regimens (39, 40). Logistic regression models were employed in several studies, particularly when the datasets had fewer predictors or were focused on well-defined clinical outcomes (42, 43, 48). In contrast to the above-mentioned studies, the study conducted by Sidonio Jr et al. in utilized a unary predictive model based on positive-unlabeled learning, which compared the characteristics of the diagnosed patient population to a potential undiagnosed population using a set of 12 key predictive variables (47).

#### Model development and optimization

The studies reviewed employed various strategies to develop and refine machine learning models for bleeding disorder prediction. A common approach was splitting datasets into training and testing subsets, often in an 80:20 or 75:25 ratio, as seen in Rawal et al., (45). Several studies incorporated cross-validation techniques to enhance model robustness (39–45, 48, 49). For example, Chowdary et al., applied repeated nested cross-validation to ensure models were trained on different subsets of data, preventing overfitting (48). Similarly, An et al., used external validation with an independent prospective cohort of 1,097 patients to test generalizability (39). Aleksic et al., adapted their validation strategy due to a small sample size (96 patients), opting for a training set method tailored to their dataset (46).

Model optimization played a critical role in improving predictive performance. Various techniques, including hyperparameter tuning (48), feature selection (45, 48), and data balancing (41), were applied. Several studies employed grid search to fine-tune hyperparameters (48). For instance, Hu et al., optimized CatBoost and random forest models using grid search to maximize accuracy and recall (40). Similarly, Rawal et al., leveraged hyperparameter tuning for LightGBM, which ultimately outperformed other models with an F1-score of ∼0.99 (45). The F1-score is a measure of a model’s balance between precision and recall, making it particularly useful for imbalanced datasets where false positives and false negatives must be minimized (51).

To address class imbalances and enhance generalizability, some studies implemented data balancing techniques. Lopes et al., used ADASYN (Adaptive Synthetic Sampling) to create a more evenly distributed dataset, preventing bias toward overrepresented classes (41). Additionally, Singh et al., explored different encoding approaches, such as One-Hot Encoding (OHE) and Position-Specific Mutation (PSM) encoding, finding that PSM improved classification accuracy for hemophilia A mutations (44).

Feature selection was another key optimization strategy. An et al., applied Lasso regression to remove redundant features while preserving the most significant predictors, improving model performance (39).

Some models incorporated genetic, molecular, and environmental data alongside clinical variables to improve prediction. For instance, Graph Neural Networks, Position-Specific Mutation encoding, and LightGBM were applied to predict disease severity, mutation effects, or inhibitor development (40, 44, 45, 49). Environmental and sociodemographic factors such as toxin exposure, smoking, diet, and comorbidities were also integrated in several models to enhance risk assessment (39, 40, 45, 46).

Model evaluation was reinforced through rigorous validation techniques, such as nested cross-validation (48), stratified k-fold validation (48), and external validation datasets (39). Performance metrics included accuracy, precision, recall, F1-score, and AUROC (Area Under the Receiver Operating Characteristic Curve). For example, Chowdary et al., reported an AUROC of 0.785 for their best-performing random forest model (48). The AUROC measures a model’s ability to distinguish between positive and negative cases, with a higher value indicating better discriminatory performance (52). However, these performance metrics should be interpreted cautiously. The retrospective design, small sample size, and lack of external validation increase the risk of overfitting and artificially inflated AUROC values. Publication bias–favoring positive results–may also overstate the clinical utility of these models.

### AI patterns in bleeding disorders

Artificial intelligence (AI) has emerged as a valuable tool in the study of bleeding disorders, facilitating risk stratification, early diagnosis, and treatment optimization. Machine learning models have been employed across various bleeding disorders, including hemophilia, von Willebrand disease (VWD), immune thrombocytopenia (ITP), and cirrhosis-related variceal bleeding, to identify predictive patterns that enhance clinical decision-making.

#### Hemophilia: predicting disease severity and treatment response

Machine Learning applications in hemophilia focused on predicting disease severity, inhibitor development, and optimizing prophylaxis. Singh et al., applied Random Forest and SVM models to mutation data, demonstrating that specific missense mutations in conserved regions of the Factor VIII gene strongly correlated with disease severity (44). Rawal et al., integrated genetic and immunological features using LightGBM to predict inhibitor development, achieving an F1-score of ∼0.99, indicating highly balanced performance (45). Ferreira et al., employed Graph Neural Networks to analyze mutation severity, achieving up to 70% accuracy based on molecular and structural data (49). Lopes et al., examined non-synonymous point mutations in FVIII protein using Decision Tree, Random Forest, and XGBoost models, validating predictions against in vitro data and clinical reports (41).

Key predictors included baseline Factor VIII activity, F8 mutation type, cumulative bleed count, and protein structure interactions (41, 45). Feature selection methods such as SHAP and Lasso regression improved model performance by removing less relevant variables (39, 48). Hu et al., analyzed a dataset of over 23,000 individuals with hemophilia, using AI models to detect undiagnosed cases based on bleeding event frequency and inhibitor development risk (40). Rawal et al., applied machine learning to predict inhibitor-negative hemophilia A status, identifying undiagnosed mutations with high precision using biologically relevant variables such as F8 mutation types and peptide-HLA binding affinities (45).

#### Von Willebrand disease: enhancing early detection and risk stratification

Machine Learning models improved early detection and classification of VWD. Sidonio Jr. et al., developed a model using Random Forest and Gradient Boosting Machines to detect von Willebrand Disease (VWD), identifying 48,902 undiagnosed cases, including 28,463 females and 20,439 males (50). This model leveraged features such as bleeding patterns, healthcare utilization, and demographic characteristics to recognize subtle indicators of VWD, achieving high accuracy (85% for males, 84% for females) (50). Heavy menstrual bleeding (HMB) in females and epistaxis in males were identified as key predictors (50). Similarly, Sidonio Jr. et al., utilized a positive-unlabeled learning approach to detect undiagnosed VWD cases, with their model achieving a positive predictive value (PPV) of 83% in the best-fit group and 75% in the good-fit group, highlighting key predictors such as the number of bleeding-related procedures and total bleeding claims (47).

The study conducted by Chowdary et al., used SHAP (Shapley Additive Explanations) values to identify the most critical predictors, such as von Willebrand factor levels and cumulative bleed count, eliminating less relevant variables (48).

#### Immune thrombocytopenia: differentiating disease subtypes and predicting clinical outcomes

Machine Learning approaches distinguished ITP from other thrombocytopenias and predicted clinical outcomes. Miah et al., developed models using demographic and hematological parameters, selecting Random Forest due to superior accuracy (100%) (43). An et al., integrated variables such as infection status, cardiovascular disease, platelet trends, diabetes, and disease duration to assess bleeding risk (39).

Key predictors included platelet count, absolute lymphocyte count, cardiovascular comorbidities, and disease duration (39, 43). ML models also enabled the identification of undiagnosed or at-risk ITP patients by analyzing electronic medical records and trends in laboratory and clinical data (39).

#### Cirrhosis and variceal bleeding: risk prediction and clinical decision support

Machine Learning classifiers predicted variceal bleeding risk based on clinical, biochemical, and endoscopic parameters. Aleksic et al., identified spleen diameter, platelet count, and the presence of large esophageal varices as the strongest predictors (46). Environmental factors such as toxin exposure and disease progression also influenced bleeding risk (46). Predictive models were applied to longitudinal data, tracking dietary intake, prophylaxis compliance, and toxin exposure to provide early warnings for high-risk patients.

### Cross-disorder predictors

Across bleeding disorders, key predictors consistently included:

- Genetic factors: F8 mutations, conserved missense mutations, non-synonymous point mutations (41, 44, 45)
- Laboratory biomarkers: Factor VIII activity, platelet count, neutrophil and lymphocyte levels (39, 43, 45)
- Clinical history: Cumulative bleed count, procedures, comorbidities, disease duration (39, 45, 47)
- Demographic factors: Age, sex, and sociodemographic/environmental exposures (40, 45, 46, 50)

**FIGURE 2:**
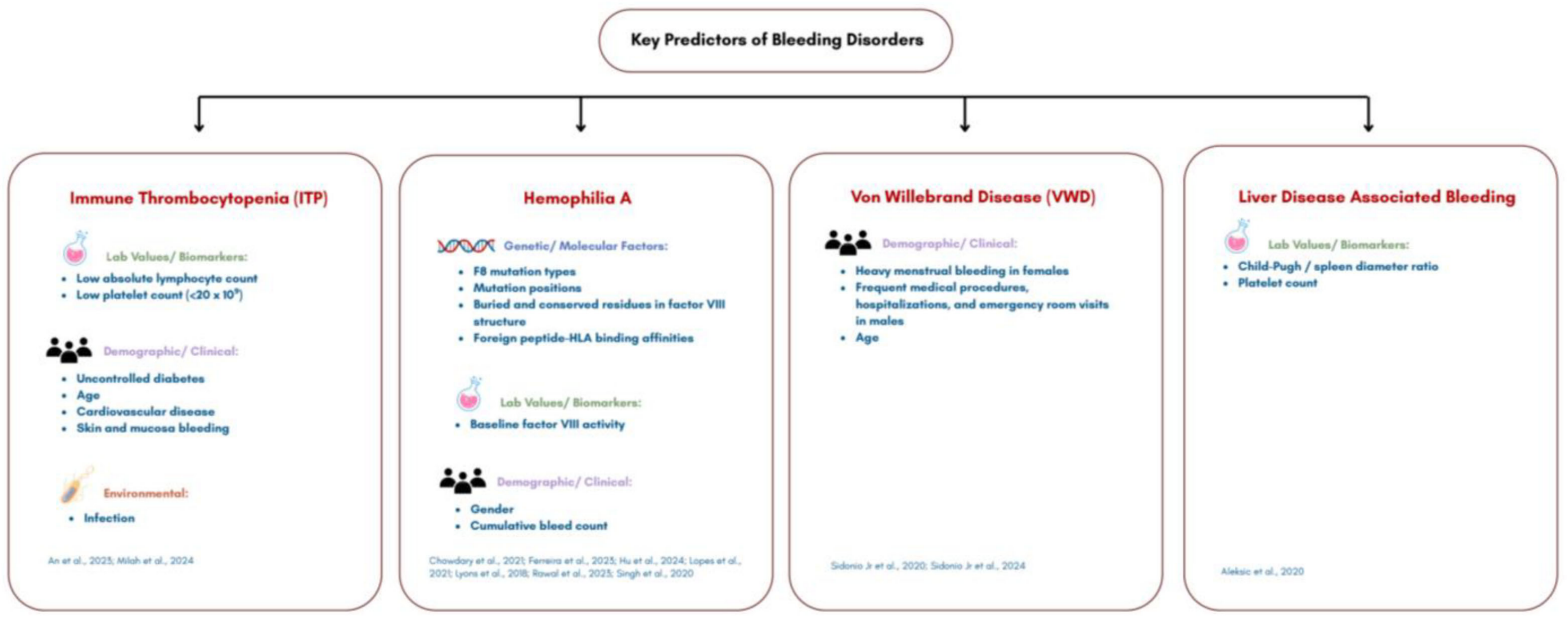
Predictors of bleeding disorders.

Integration of these predictors into ML algorithms enhanced disease classification, early diagnosis, and personalized treatment strategies (Figure 2).

## Discussion

This systematic review highlights the advancements in machine learning (ML) applications for predicting and managing bleeding disorders. A comparison of the best-performing models across studies provides valuable insights into their predictive performance, strengths, and limitations.

### Comparison of best-performing models

Across studies, Random Forest, XGBoost, and LightGBM consistently demonstrated strong predictive performance and interpretability. For immune thrombocytopenia (ITP), Random Forest achieved high accuracy, particularly in distinguishing ITP from other thrombocytopenic conditions. In hemophilia A prediction, LightGBM and Random Forest were most effective, with LightGBM achieving an F1-score of ∼0.99. Gradient Boosting Machines and Random Forest also performed well in von Willebrand disease (VWD) classification, identifying undiagnosed cases with high accuracy. For cirrhosis-related variceal bleeding, LogitBoost proved highly accurate, while broad population-level analysis favored Random Forest and CatBoost for target joint prediction in hemophilia patients.

### Strengths and limitations

The success of ensemble learning models underscores their ability to handle complex, multidimensional datasets. Techniques such as SHAP values and Lasso regression improved interpretability, while hyperparameter tuning enhanced predictive performance. Nonetheless, several limitations were evident across the literature. Common issues included class imbalance, missing data, and confounding, all of which may have influenced reported outcomes. External validation was limited, constraining generalizability. Deep learning models, although promising, demonstrated only moderate accuracy and remain difficult to implement clinically due to their “black box” nature. Addressing these limitations will require improved dataset quality, broader external validation, and the development of interpretable models suited for real-world settings.

The outcomes predicted by AI models also varied considerably. Short-term predictions included critical bleeding within weeks (An et al.) and treatment response over 12 weeks (Chowdary et al.). Long-term predictions encompassed inhibitor development, target joint formation, and severity classification. This range suggests that some models are better suited to acute decision-making, while others inform longitudinal management. Most studies relied on baseline predictors–demographics, genetics, or initial laboratory values–while only a few incorporated time-varying variables such as serial platelet counts, evolving comorbidities, or treatment adherence. Incorporating longitudinal data could substantially enhance clinical relevance.

Although a meta-analysis could have pooled diagnostic accuracy estimates, the heterogeneity of model architectures, predictors, outcomes, and metrics made statistical synthesis inappropriate. Instead, standardized reporting of AUROC, PPV, calibration, and consistent outcome definitions will be essential for future meta-analyses. Importantly, several studies were rated as moderate to high risk of bias–particularly due to missing outcome data, inadequate adjustment for confounders, or selective reporting–likely inflating reported accuracy. Conversely, findings from low-risk studies (Sidonio Jr et al.) more likely reflect true clinical performance. Overall, while results are promising, confidence is stronger in models with rigorous methodology and external validation.

Despite encouraging technical results, adoption remains slow. Challenges include fragmented datasets, poor interoperability across IT systems, regulatory hurdles, limited clinician familiarity, and concerns over transparency. Access inequities persist, especially in low-resource settings. Addressing these barriers will require coordinated efforts–multi-center registries, integration of interpretable AI into EHRs, and clinician training.

### Heterogeneity and generalizability

Heterogeneity was evident across disorders and study designs. In hemophilia, genetic and structural features dominated; in VWD, healthcare utilization and bleeding patterns were key; in ITP, platelet counts and comorbidities were critical; and in cirrhosis, clinical and endoscopic data were central. Shared predictors such as platelet count and factor VIII activity emerged across disorders. However, most models were developed in high-income countries, limiting insights for low-resource contexts where diagnostic gaps are greatest. Future work should test generalizability in these settings.

### Clinical implications and future directions

Machine learning approaches show promise for early diagnosis, risk stratification, and treatment optimization in bleeding disorders. Identifying undiagnosed cases–particularly in VWD and hemophilia–highlights potential for improved outcomes. Yet, most studies remain confined to retrospective design and internal validation. Prospective validation, randomized controlled trials of AI-assisted decision-making, and cost-effectiveness analyses are needed to guide real-world adoption.

Implementation should emphasize metrics with direct clinical meaning. For instance, PPV reflects the proportion of positive predictions that are correct and is critical in bleeding disorders, where false positives may lead to unnecessary factor replacement, invasive procedures, or prolonged monitoring. Sidonio Jr et al., reported PPVs of 75%–83%, which are encouraging but still insufficient for routine practice.

Practical integration requires embedding tools into clinical workflows. Examples include EHR-based risk alerts in ITP or dosing support in hemophilia prophylaxis. Barriers include interoperability, trust, regulation, and cost. Solutions may involve federated learning for data privacy, decision-support dashboards for clinicians, and structured training.

Future research should prioritize prospective, multi-center validation, registry-based adaptive trials, and integration of longitudinal and environmental data. Wearables and remote monitoring could enable personalized, real-time management. At the policy level, regulators must establish frameworks for AI adoption in rare hematologic disorders, ensuring safety while encouraging innovation.

## Conclusion

Machine learning has emerged as a transformative tool in bleeding disorder prediction and management, enabling early diagnosis and personalized treatment. Ensemble models such as Random Forest, XGBoost, and LightGBM demonstrate strong predictive capabilities, but challenges like data imbalance and limited external validation must be addressed for broader clinical adoption.

Future research should focus on real-world validation, electronic health record integration, and explainable AI methodologies. By refining ML models and ensuring their accessibility in clinical workflows, these advancements can enhance bleeding disorder management, ultimately improving patient outcomes and quality of life.

## Data Availability

Publicly available datasets were analyzed in this study. This data can be found here: Will be available on request.
